# Detection of DNA Viruses in Free-Ranging Rat Populations in Hungary

**DOI:** 10.3390/v16121948

**Published:** 2024-12-19

**Authors:** Márton Z. Vidovszky, András Surján, Gábor Földvári, László Egyed

**Affiliations:** 1 HUN-REN Veterinary Medical Research Institute, 1143 Budapest, Hungary; vidovszky.marton@vmri.hun-ren.hu (M.Z.V.); surjan.andras@vmri.hun-ren.hu (A.S.); 2 Institute of Evolution, HUN-REN Centre for Ecological Research, 1121 Budapest, Hungary; 3 Centre for Eco-Epidemiology, National Laboratory for Health Security, 1077 Budapest, Hungary

**Keywords:** free-ranging *Rattus norvegicus*, *Rattus rattus*, PCR, adenoviruses, herpesviruses, polyomaviruses, circoviruses

## Abstract

To address a gap in our understanding of viral infections in epidemiologically important rat species, we aimed to detect DNA viruses from the tissues of free-ranging rat populations in Hungary. DNA viruses were identified from the parenchymal organs of 230 *Rattus norvegicus* and *Rattus rattus*, using family-specific pan-PCR assays followed by sequencing of the PCR products. Adeno-, herpes-, circo-, and polyomaviruses were detected, while irido-, pox-, and dependoparvoviruses were not. Adenovirus DNA was present in 6.5% of the samples, herpesvirus and polyomavirus DNA in 12.2%, and circovirus DNA in 1.7%. All detected herpesviruses belonged to the β and γ subfamilies, with a majority being β herpesviruses. Some adenovirus and herpesvirus sequences were novel, while only the known Rattus norvegicus polyomavirus 1 was detected for polyomaviruses. The rare circovirus-positive samples revealed the presence of both rodent and bird circoviruses, indicating the ability of circoviruses to cross species barriers. Our findings show that rats host a variety of DNA viruses, many of which were previously uncharacterized, highlighting the need for further diagnostic studies.

## 1. Introduction

Rodentia is the largest and most diverse group of mammals, comprising over 2500 species and representing 43% of all mammalian species. With a wide range of structural and functional adaptations, rodents successfully occupy nearly every terrestrial habitat and are frequently found in close association with humans and domestic animals. This is especially true for *Rattus norvegicus* and *Rattus rattus*. Although *R. norvegicus* still exists in the wild in parts of the Far East, such as Manchuria and Korea, close to the geographic area where the species originated [1], its populations are far more abundant in urban areas and human settlements. Their proximity to human environments and attraction to human food and waste have drawn the attention of researchers to their potential role as reservoirs of zoonotic viral pathogens.

While free-ranging sewer and field rats are often the focus of epidemiological and microbiological studies [2,3], most of these surveys have largely concentrated on bacterial zoonotic pathogens. Although the majority of human pathogens are zoonotic, our understanding of the viral diversity in rats remains limited, particularly for DNA viruses such as herpesviruses (HVs), polyomaviruses (PyVs), poxviruses, and parvoviruses, which have rarely been isolated from wild rats [4,5,6,7]. Despite ongoing efforts, the knowledge of DNA viruses carried by rat species remains insufficient.

Previous studies of non-rat rodent species have provided more insights into viral infections. For example, a large-scale microbiome study of over 3000 rodents in China detected HV, adenovirus (AdV), and circovirus sequences in various rodents, although the impact on rat species is unclear [8]. Natural AdV infections have been described in other rodents, such as laboratory mice (murine adenovirus 1 (MadV-1) and -2) [9] and free-ranging rodents like the striped field mouse (*Apodemus agrarius*—MAdV-3) [10], and from a wide range of free-ranging rodent species (mice and shrews) of the African continent [11], although AdVs were not published from any rat species. Similarly, HV strains belonging to the *Betaherpesvirinae* (β) subfamily were isolated from *R. norvegicus* and *R. rattus* [12,13], while HVs belonging to the *Gammaherpesvirinae* (γ) subfamily were detected from five rat species [14,15].

There is limited knowledge of circoviruses in rats, with circovirus sequences being found in several rodent species [8], like the smoke-bellied rat (*Niviventer eha*). Another complete rat circovirus genome was deposited in GenBank (MF497827, NC055121) from the hoary bamboo rat (*Rhizomys pruinosus*), belonging to a different family (Spalacidae) from rats (Muridae), captured in South China. These viruses are known to jump species barriers [16,17], potentially posing an epidemiological risk in animal husbandry [18,19], and rats may serve as reservoirs for such viruses. Meanwhile, scattered data suggest that DNA viruses can infect rats [20].

Similar to the viruses mentioned earlier, our knowledge of rat PyVs is still limited, although 24.5% of 421 *R. norvegicus* from five European countries were found to carry rat PyVs [3].

Poxvirus infections are not too frequent in rats, although important non-rodent poxviruses, like cow- and monkeypox infections, occur in them [21,22]. A poxvirus—not characterized in detail, probably an orthopoxvirus—was isolated from the tail lesion of a *R. norvegicus* individual in Kuwait [6].

In contrast to the extensive research focusing on rodents’ prominence as known reservoirs of zoonotic viruses in general, there has been relatively less emphasis on rat species. The scattered data of DNA virus detections of individuals of rare rat species indicate the susceptibility of rats for DNA virus infections, but reliable data from the most abundant and epidemiologically important species, *R. norvegicus* and *R. rattus*, are still lacking. This study aimed to detect DNA viruses in rats using family-specific PCR assays, providing a relatively large number of rats in order to obtain a more detailed picture of the DNA viruses present in rat populations and their potential epidemiological roles in maintaining and spreading infections.

## 2. Materials and Methods

### 2.1. Sample Collection and Preparation

**Figure 1:**
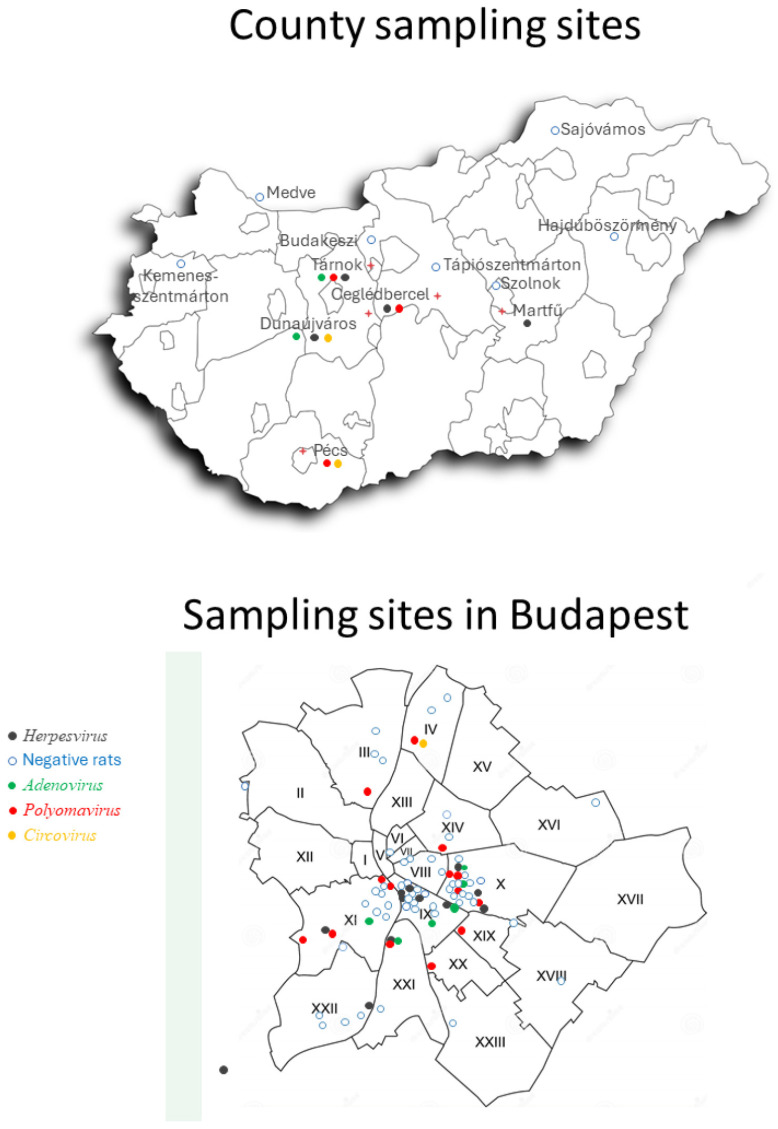
Sampling sites on a map of Budapest and Hungary. The viral positivity of samples from the locations is indicated by different colors. The location of the sampled cities is indicated by a red cross.

Tissues from 230 poisoned rats (214 *R. norvegicus*, 93%, and 16 *R. rattus*, 7%) were used as samples. All animals were fresh (<24 h), and the resected tissues were kept frozen before processing. A total of 149 rats (64.8%) came from Budapest, with the remaining 81 (35.2%) from 11 other settlements in Hungary (towns, villages, livestock farms, zoos, and Danube harbor, Figure 1).

Data on body size (grams, cm), gender, capture/death site, and time were recorded. The main visceral organs (spleen, liver, kidneys, lungs, heart, brain, and salivary glands) were aseptically resected. Cell suspensions from these organs were made in distilled water, and DNA was extracted from these suspensions using the Genomic DNA Mini Kit (Geneaid Biotech Ltd., New Taipei City, Taiwan). One µL of the eluted DNA was used as a template in subsequent PCR assays.

### 2.2. PCR Assays

In all PCR assays, we used the same chemicals, enzymes, and kits. The 20 μL reaction volume of the PCR mix contained the following: 10 μL of DreamTaq Hot Start PCR Master Mix (ThermoFisher Scientific™ Baltics UAB, Vilnius, Lithuania), 0.5 μL of each primer (10 pmol/μL), 1 μL of template DNA, and 8 µL of Milli-Q water. Amplifications followed the cycling parameters described in the publications shown in Table 1. Only Hanson’s method [23] was a simple PCR (a robust assay for detecting various large DNA viruses, such as pox-, herpes-, iridoviruses, and AdVs), while the others were all nested PCRs. In the second round of nested PCRs, 1 μL of the first PCR product was used as a template. Amplicons were visualized by agarose gel electrophoresis.

### 2.3. DNA Sequencing

PCR products were isolated and purified using the NucleoSpin^®^ Extract II kit (Macherey-Nagel GmbH & Co., Düren, Germany). Sequencing was performed with the inner primers from the nested PCRs on both strands using the BigDye™ Terminator Cycle Sequencing Kit (Applied Biosystems, Waltham, MA, USA). Capillary electrophoresis was conducted on an ABI Prism 3500 Genetic Analyzer by a commercial scientific service.

### 2.4. Sequence Analysis

Sequence identity was determined using the blastx and blastn algorithms on the online BLAST platform (https://blast.ncbi.nlm.nih.gov/Blast.cgi, accessed on 24 July 2024). Homologous nucleotide sequences were aligned using the MultAlin program (http://multalin.toulouse.inra.fr/multalin/, accessed on 24 July 2024). Amino acid sequences were verified with the ExPAsy translate tool (https://web.expasy.org/translate/, accessed on 24 July 2024).

### 2.5. Phylogenetic Analysis

Homologous nucleotide sequence-parts of the DNA polymerase genes from AdVs and HVs alike were aligned using MAFFT 7.49 with the FFT-INS-i algorithm [29]. The multiple sequence alignment was refined using trimAI 1.4.1, with automatic parameters [30]. Refined AdV sequences were 273 nt long, while βHV and γHV sequences were 740 and 688 nt long, respectively. The phylogenetic tree reconstruction based on the DNA polymerase nucleotide sequences was performed by PhyML 3.3.1. [31], using smart model selection (SMS) [32] with BIC. The best model was the GTR + G + I substitutional model, the proportion of invariant sites and the parameter for the gamma model were estimated, while equilibrium frequencies were determined empirically. Subtree Pruning and Regraphing (SPR) was applied for tree topology searches. Branch support values were calculated with an SH-like aLRT method [31].

## 3. Results

### 3.1. Adenoviruses

**Figure 2:**
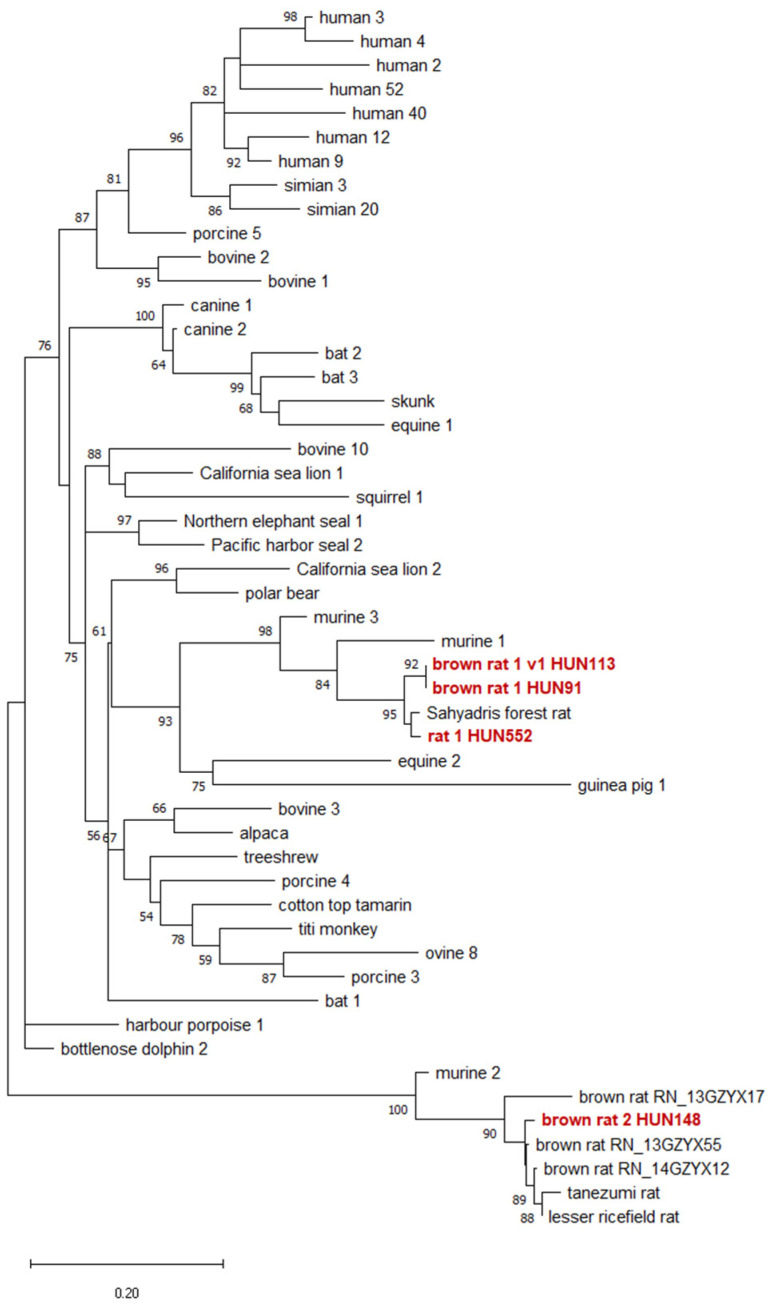
Phylogenetic tree of mastadenoviruses. The designation ‘adenovirus’ was omitted from the virus names; the Maximum Likelihood tree is based on the short nucleotide sequence of the DNA polymerase gene. SH-like aLRT support values of the branches are shown at the nodes. Our novel rat AdVs are indicated with bold red names. The bar represents 0.2 nucleotide substitutions per site.

Fifteen samples tested positive for AdV (6.5%), two of which were identical to bovine adenovirus 2 (BAdV-2), and five of which were identical to a human AdV of the *Mastadenovirus caesari* species (formerly *Human mastadenovirus C*). From the remaining eight samples, three different AdVs were detected (with one AdV showing two nucleotide variants). These sequences shared 92–98% aa identity with different rodent adenoviruses in GenBank (Table 2). One of the detected AdVs was named rat AdV-1, as it was detected in both *R. norvegicus* and *R. rattus*. Phylogenetic studies revealed that the newly discovered rat AdVs formed two well-separated and distinct phylogenetic clades with previously known rodent AdVs. One of the two separated groups follows the well-known distinct evolutionary path of MAdV-2, while the other forms a monophyletic branch with MAdV-1 and -3 (Figure 2). Some of the novel AdVs showed very small genetic distance from each other. The novel brown rat AdV-2 belonged to a clade with other already described *R. norvegicus* AdVs.

### 3.2. Herpesviruses

Twenty-eight samples (12.2%) were positive for HVs. Eight of these samples carried DNA polymerase gene fragments of two HV types (6, 2) from the *Gammaherpesvirinae* subfamily. Both were identical to previously known gammaherpesviruses (γHVs), with 100% aa identity (Table 2). Twenty (71%) of the HV+ sequences belonged to the β subfamily (*Betaherpesvirinae*), with aa similarity ranging from 86% to 99%. Four different betaherpesviruses (βHVs) have been detected, two of which belonged to the species *Muromegalovirus muridbeta8*, one to *Muromegalovirus muridbeta2*, and one was presumably a representative of a new species. We detected two variants of both HVs of the species *M. muridbeta8* that do not differ in aa, only on a nucleotide level. No members of the *Alphaherpesvirinae* subfamily were detected from the examined rats (Table 2).

**Figure 3:**
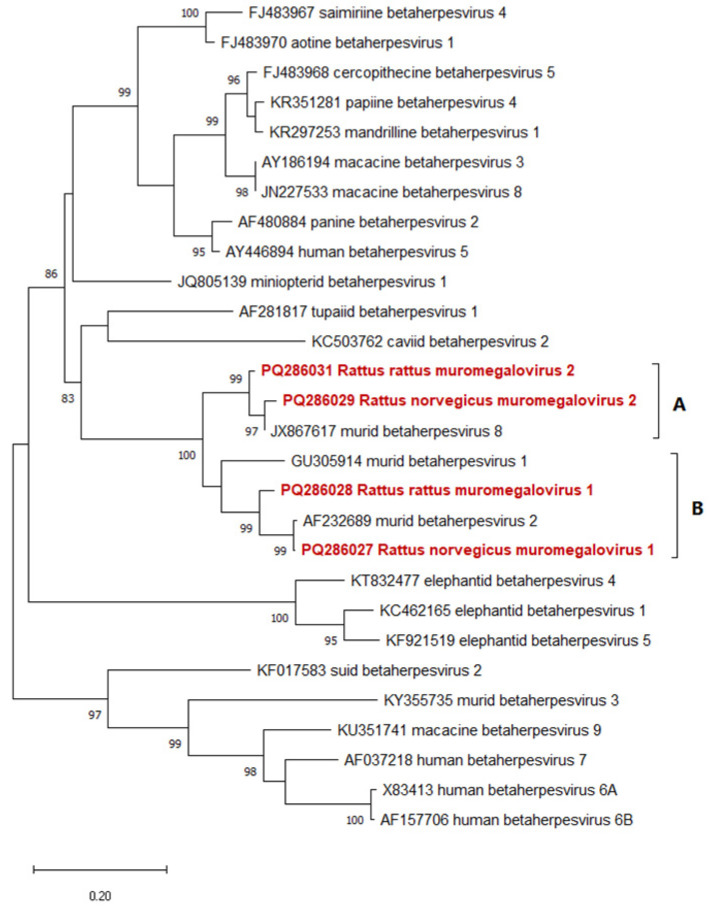
Phylogenetic tree of betaherpesviruses. The Maximum Likelihood tree is based on the short nucleotide sequence of the DNA polymerase catalytic subunit. SH-like aLRT support values of the branches are shown at the nodes. Our novel rat βHVs are indicated with bold red names. The bar represents 0.2 nucleotide substitutions per site. Subgroup “A” represents the members of the species *Muromegalovirus muridbeta 8* while subgroup “B” contains members of several betaherpesvirus species just like *Muromegalovirus muridbeta 1-2*, and a virus that possibly represent a novel betaherpesvirus species.

On the phylogenetic tree, in regard to the rat βHVs reported in this study, all belonged to a common large clade with Murid betaherpesvirus 1, —2, and —-8, although the clade could be divided into two subgroups. Two of the newly detected rat βHVs were clustered together in subgroup A, with small evolutionary distances between them. This subgroup contained Murid betaherpesvirus 8. The other novel βHVs belonged to the subgroup B with Murid betaherpesvirus 2 and showed larger evolutionary distances from one another (Figure 3).

**Figure 4:**
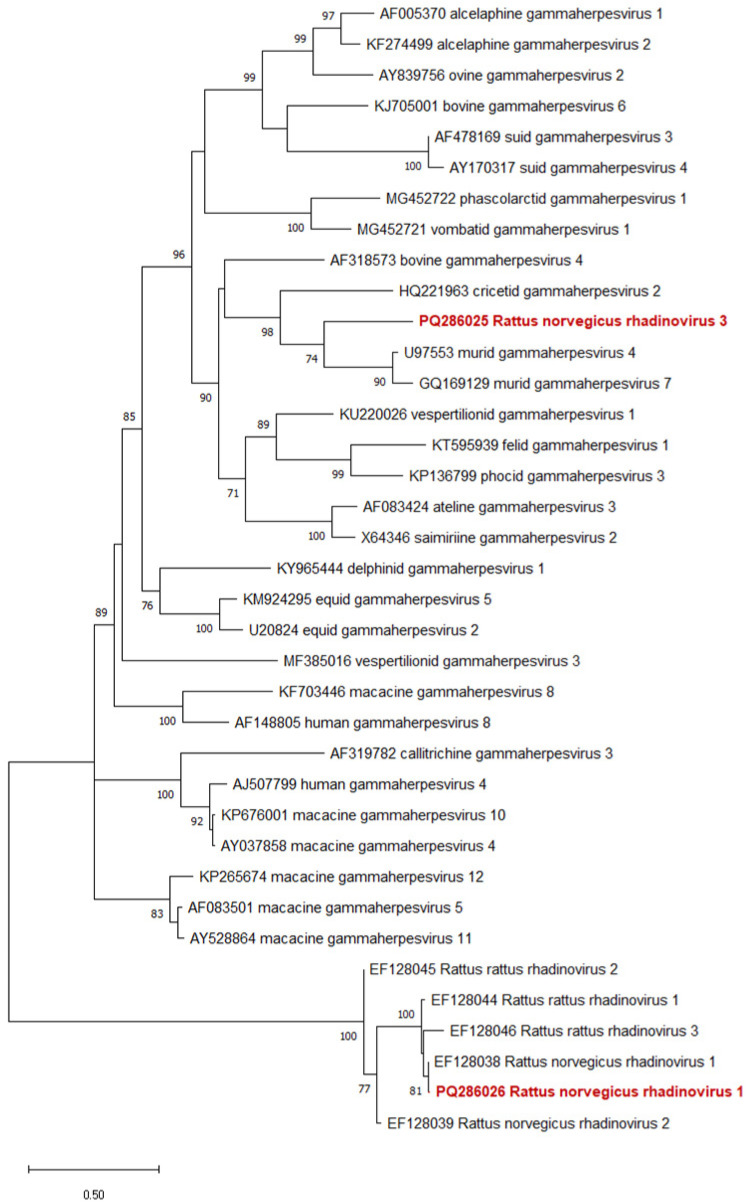
Phylogenetic tree of gammaherpesviruses. The Maximum Likelihood tree is based on the short nucleotide sequence of the DNA polymerase catalytic subunit. SH-like aLRT support values of the branches are shown at the nodes. Our novel rat γHVs are indicated with bold red names. The bar represents 0.5 nucleotide substitutions per site.

The novel γHV sequences belonged to two different groups (Figure 4). Both belonged to subgroups with other rodent γHVs. Conversely, Rattus norvegicus rhadinovirus 1 was a member of a subgroup with other γHVs that are also derived from rat hosts and genetically closely related. Rattus norvegicus rhadinovirus 3 is the member of a subgroup with γHVs from other, non-rat rodent hosts.

### 3.3. Circoviruses

Four samples tested positive for circoviruses (Table 2). Sequencing of organ samples from two rats showed the presence of pigeon circovirus, while two other samples revealed circovirus variants recently detected in an owl, but it was later published as presumed to be originally a rat circovirus [33].

### 3.4. Polyomaviruses

We detected Rattus norvegicus polyomavirus 1 sequences in 28 samples (12.2%). Most of the sequences (26) were 100% identical to each other on the nucleotide level (deposited to the GenBank with the accession number: PQ261247), while being also 100% identical on the amino acid level to the Rattus norvegicus polyomavirus 1 (NC_027531.1). The sequences from the other two samples (PQ261248 and PQ261249) showed single point mutations on the 220 bp long amplified sequence part. Only one of the point mutations (in PQ261249) led to an amino acid change (valine to alanine).

## 4. Discussion

This study expands our understanding of the viral families examined. Most of the sequence data indicated that we found previously undetected viruses in rats (adeno-, herpes-, circo-). However, we also identified known rodent viruses (adeno-, herpes-, polyoma-). The only nested PCR that did not yield positive results was the one targeting dependoviruses [28]. Nested PCR appears to be more sensitive, as the used simple PCR [23] targeting AdVs, along with other large DNA viruses [23], was negative in all samples, while the nested AdV PCR [24] showed positive results in several cases. BAdV-2 and human AdVs of the *Mastadenovirus caesari* species were detected in rat samples. The detection could be linked to the rats’ lifestyle, possibly through sewage exposure. This result is not unexpected, as BAdV-2 belongs to the same species as AdVs from other host species (ovine AdVs), and AdVs from the *Mastadenovirus caesari* species are known to replicate in various host cells, including rodents [34]. All the rodent AdVs detected in this study are likely novel. However, due to their low percentage of sequence divergence from known rodent AdVs in the GenBank (2–8%), they probably do not represent new species but may belong to the same species as their closest relatives. Further genome sequencing will confirm their exact classification. The AdVs were named according to the host species from which they were detected, following the established method. One of the described AdVs was named rat AdV-1, as it was found in both *R. norvegicus* and *R. rattus*. Despite the low aa difference between the detected rat AdV-1 and brown rat AdV-1 (95% identical to each other, 96% and 92% identical to an AdV detected in *Rattus satarae* in India), there were substantial nucleotide (nt) differences (15–18% difference), which indicates the presence of many silent mutations. This may indicate that these viruses are ancestral AdVs that have co-evolved with rat hosts for a very long time, more or less preserving the protein structure of the very conserved DNA polymerase gene.

The rat AdVs we detected were clearly grouped with previously known rodent AdVs. While it is known that murine adenovirus 2 (MAdV-2) represents a distinct evolutionary lineage compared to murine adenoviruses 1 and 3 (MAdV-1, MAdV-3) [9], two of the newly detected rat AdVs (brown rat AdV-1 and rat AdV-1) surprisingly show a relationship with MAdV-1 (*Mus musculus*) and MAdV-3 (*Apodemus agrarius*). In contrast, previously known rat adenoviruses detected in the same host, *R. norvegicus*, and from other species, are located in a separate clade with MAdV-2 and with one of our AdV sequences (brown rat AdV-2) (Figure 2).

In the case of βHVs, we detected altogether four HVs. Two (Rattus rattus muromegalovirus 2 and Rattus norvegicus muromegalovirus 2) formed subgroup A with murid betaherpesvirus 8 (MβHV-8) and two (Rattus rattus muromegalovirus 1 and Rattus norvegicus muromegalovirus 1) formed subgroup B with murid betaherpesvirus 2 (MβHV-2). In both subgroups, one was detected only from *R. norvegicus* and the other from *R. rattus*, suggesting the species specificity of these HVs (Figure 3). The two newly detected MβHV-2-like HVs were detected in only three samples. One belonged to the *Muromegalovirus muridbeta2* species, and the other may represent a new species, which requires further sequence data to confirm (Rattus rattus muromegalovirus 1). HVs similar to MβHV-8 were detected in many more samples (12 + 5), likely belonging to the *Muromegalovirus muridbeta8* species. In these cases, we also detected nt variants with silent mutations on the studied ~250 nt long sequence part (Table 2). These latter findings suggest that HVs belonging to the *M. muridbeta8* species may be evolutionarily older in rats, having accumulated numerous silent mutations over time.

For γHVs, Rattus norvegicus rhadinovirus 1 was detected in six samples and clustered with other closely related rhadinoviruses from rat hosts. Although this group included rhadinoviruses from various rat species, its phylogenetic distance from other viruses makes determining their origin challenging. Rattus norvegicus rhadinovirus 3 was detected in only two samples and is a member of a subgroup with γHVs from other rodent host species. This suggests that perhaps the above-mentioned clade, containing Rattus norvegicus rhadinovirus 1 and 2, may represent the original γHVs of rats, whereas Rattus norvegicus rhadinovirus 3 could have originated from non-rat rodent hosts (Figure 4). The newly detected Rattus norvegicus rhadinovirus 3 is so distant from other known γHVs that it is presumably a novel rhadinovirus, and may represent the first member of a novel species. Here again, further sequencing of the genome is needed to confirm this. γHV was detected exclusively in *R. norvegicus*, with no γHV found in *R. rattus* samples.

Our herpesvirus results align with previous similar studies in many ways, but some differences were observed. In Central Africa, a much lower prevalence of herpesviruses was reported, with only 2 out of 1024 free-ranging wild rodents and shrews testing positive for herpesviruses (3.45%) [15]. Both rats carried γHV sequences (Bandicota indica rhadinovirus 1 and Rattus norvegicus rhadinovirus 2). A German study that analyzed 1132 rodent tissue samples found a herpesvirus prevalence of 26.6%, including 19.34% γHVs and 7.24% βHVs [14]. The prevalence in our study was lower compared to the German study, but the results are limited due to differences in sample numbers, storage, and processing. We could not detect αHVs from our samples, similarly to the authors of the two previously cited papers. Our results differ from the literature data in that the large majority, notably 71% of our sequences, were βHVs, which is unusual since βHVs are typically less frequent in mammals, in contrast with the members of α and γ subfamilies. The cause of the lack of αHVs and the abundance of βHVs is not easy to answer. The organ samples we used for DNA extraction contained the large parenchymal organs and salivary glands. Salivary glands are the main latency site of βHVs, which could be a reason for the high prevalence of βHVs in our samples.

The relatively few circovirus-positive samples indicate that circoviruses do not cause widespread infections in rat populations. The rare detection of pigeon circovirus sequences in rats can be explained, as circoviruses are known to jump species barriers, as seen with swine circoviruses in rats [18,35], and urban rats could have direct contacts in their common environment to both pigeon carcasses and their feces. Due to the demanding nature of laboratory work and the fact that we tested samples of internal organs, the contamination of samples can be excluded. The other two circoviruses detected were very similar to a virus recently isolated from a tawny owl [16]. Based on their position on the phylogenetic tree (presented in a previous study [33]) and their occurrence in the tissues of rats, these were probably the genuine circovirus species of rats, or of a closely related rodent species [36]. One of the circovirus-positive rats was a *R. rattus*, indicating susceptibility to pigeon circovirus in both rat species.

In our samples, only very similar variants of a single rodent PyV were found. The PyV positivity rate of our samples (12.2%) was slightly lower than previously observed [3]. With one exception, the detected PyV sequences were aa identical to the Rattus norvegicus polyomavirus 1 found in GenBank. The same PyV was also detected in *R. rattus*, indicating susceptibility of both rat species to infection by this PyV. Therefore, we named the detected virus Rattus polyomavirus 1, although variants of this virus have also been detected in other rodent (*Apodemus flavicollis*, MG654478) and bat (*Pipistrellus pipistrellus*, LC426510) hosts. In 2 of the 28 positive samples from *R. norvegicus*, we detected variants differing by a single nt, one of which caused an aa shift.

We could not detect pox-, irido-, or dependoparvoviruses, which may have epidemiological and diagnostic reasons. As the literature suggests, these viruses are infrequent in rats, with only one study reporting poxvirus isolation from a skin lesion in *R. norvegicus* [5]. From a diagnostic perspective, it is important to note that only nested PCR systems were able to detect viral DNA in rat tissues, while simple PCR assays, such as those used by Hanson et al., were unsuccessful. The sensitivity of these simple assays may be insufficient for amplifying low amounts of viral target DNA, and we suggest avoiding them in similar experimental future studies with diagnostic aims.

Although only 16 *R. rattus* (6.95%) were tested, we detected pigeon circovirus (1), rat PyV (2), and murine-like βHVs 2 and 8 (5), suggesting that both rat species are equally susceptible to these viruses.

Seven suckling rats (7–8 cm, 13–16 g) from two nests (four *R. norvegicus*, three *R. rattus*) were negative for all studied viruses. As these animals lived in the nests and fed on milk, they could have been infected only through transplacental, lactogen, or salivary ways. Such infection routes were described in cases of human but not murine βHVs (cytomegalo-) [36] and human (HHV-8) and bovine (BoHV-4) rhadino γHVs [37,38]. Even if their number is low, our data could be a mild indication that DNA (herpes) viruses probably do not frequently infect young rat individuals via these pathways, or do so inefficiently.

In our study, we focused on visceral organs as primary targets, as these tissues serve as the main replication sites for DNA viruses, with the exception of parvoviruses. However, a comprehensive pan-spectrum nested PCR assay for parvoviruses has not yet been established. We deliberately excluded intestinal tissues and feces from our analysis, as these samples often contain inhibitory molecules and are predominantly associated with RNA virus replication. Nevertheless, diagnostic investigations using these tissues are feasible [39], and DNA viruses are occasionally detected in them [40]. Future studies could benefit from including intestinal tissues in the screening process for DNA viruses.

Given the large number of rats in urban environments, it is crucial to better understand the viruses they harbor in greater detail. The relatively high proportion of viruses identified at the family or genus level (primarily HVs and AdVs) in this study highlights the need and possibility to isolate and identify novel viral agents in rats during further similar studies in the future. This research could enhance our understanding of the potential new zoonotic viruses harbored by these important urbanized mammals.

## Figures and Tables

**Table 1:** Details of the applied PCR assays.

Target Virus			PCR	
Family	Gene	Authors	Type	Product Size (bp)
Adeno	DNA pol	Wellehan et al., 2004 [24]	nested	320
Herpes	DNA pol	VanDevanter et al., 1996 [25]	nested	215–315
Circo	Rep	Halami et al., 2008 [26]	nested	350
Polyoma	VP1	Johne et al., 2005 [27]	nested	210–230
Pox,	DNA pol	Hanson et al., 2006 [23]	single	570–630
Irido	DNA pol	Hanson et al., 2006 [23]	single	662–695
Parvo	Capsid	Pénzes and Benkő, 2014 [28]	single	600
(dependo)				

**Table 2:** DNA viruses detected in the study, their taxonomic classification, and the closest related GenBank sequences.

	Virus Name	Species	nr. of Samples	Host	acc. nr.	Comparison		
						Identity	Virus Name in GenBank	acc. nr.
	***Herpesvirales; Orthoherpesviridae***					aa		
	***Gammaherpesvirinae; Rhadinovirus***							
1.	Rattus norvegicus rhadinovirus 1	not classified	6	*Rattus norvegicus*	PQ286026	100%	‘Rattus norvegicus rhadinovirus 1’	EF128038
2.	Rattus norvegicus rhadinovirus 3	not classified	2	*Rattus norvegicus*	PQ286025	100%	‘Rat herpesvirus’	KX987266
	***Betaherpesvirinae; Muromegalovirus***							
3.	Rattus norvegicus muromegalovirus 1	*Muromegalovirus muridbeta2*	2	*Rattus norvegicus*	PQ286027	99%	‘Murid betaherpesvirus 2’	OP429144
4.	Rattus rattus muromegalovirus 1	possibly new	1	*Rattus rattus*	PQ286028	86%	‘Murid betaherpesvirus 2’	OP429144
5.	Rattus norvegicus muromegalovirus 2	*Muromegalovirus muridbeta8*	7	*Rattus norvegicus*	PQ286029	95%	‘Murid betaherpesvirus 8’	NC_019559
	Rattus norvegicus muromegalovirus 2 v1	*Muromegalovirus muridbeta8*	5	*Rattus norvegicus*	PQ286030	95%	‘Murid betaherpesvirus 8’	NC_019559
6.	Rattus rattus muromegalovirus 2	*Muromegalovirus muridbeta8*	3	*Rattus rattus*	PQ286031	93%	‘Murid betaherpesvirus 8’	NC_019559
	Rattus rattus muromegalovirus 2 v1	*Muromegalovirus muridbeta8*	2	*Rattus rattus*	PQ286032	93%	‘Murid betaherpesvirus 8’	NC_019559
	***Adenoviridae; Mastadenovirus***							
1.	brown rat adenovirus 1	not classified	2	*Rattus norvegicus*	PQ273847	92%	‘rodent adenovirus’ (*Rattus satarae*)	OR906164
	brown rat adenovirus 1 v1	not classified	3	*Rattus norvegicus*	PQ273848	92%	‘rodent adenovirus’ (*Rattus satarae*)	OR906164
2.	brown rat adenovirus 2	not classified	1	*Rattus norvegicus*	PQ273850	98%	Rattus norvegicus adenovirus’	KU258165
3.	rat adenovirus 1	not classified	2	*Rattus* sp.	PQ273849	95%	‘rodent adenovirus’ (*Rattus satarae*)	OR906164
4.	bovine adenovirus 2	*Mastadenovirus bovidae*	2	*Rattus norvegicus*	-	100%	‘bovine adenovirus type 2’	NC_002513
5.	human adenovirus	*Mastadenovirus caesari*	5	*Rattus norvegicus*	-	100%	‘human adenovirus type 1’	AF534906
	***Polyomaviridae; Alphapolyomavirus***							
1.	Rattus polyomavirus 1	*Alphapolyomavirus ranorvegicus*	26	*Rattus* sp.	PQ261247	100%	‘Rattus norvegicus polyomavirus 1’	NC_027531
	Rattus norvegicus polyomavirus 1 v1	*Alphapolyomavirus ranorvegicus*	1	*Rattus norvegicus*	PQ261248	100%	‘Rattus norvegicus polyomavirus 1’	NC_027531
	Rattus norvegicus polyomavirus 1 v2	*Alphapolyomavirus ranorvegicus*	1	*Rattus norvegicus*	PQ261249	100%	‘Rattus norvegicus polyomavirus 1’	NC_027531
	***Circoviridae; Circovirus***							
1.	pigeon circovirus	*Circovirus pigeon*	2	*Rattus* sp.	-	100%	Columbid circovirus	AF252610
2.	brown rat circovirus 1	not classified	1	*Rattus norvegicus*	OR553090	100%	towny owl-associated circovirus	OL411978
3.	brown rat circovirus 2	not classified	1	*Rattus norvegicus*	OR553091	100%	towny owl-associated circovirus	OL411978

## Data Availability

The original contributions presented in the study are included in the article; further inquiries can be directed to the corresponding author.
